# Risk of suicide for individuals reporting asthma and atopy in young adulthood: Findings from the Glasgow Alumni study

**DOI:** 10.1016/j.psychres.2014.12.012

**Published:** 2015-02-28

**Authors:** Andrew A. Crawford, Bruna Galobardes, Mona Jeffreys, George Davey Smith, David Gunnell

**Affiliations:** a School of Social and Community Medicine, University of Bristol, Bristol BS8 2BN, UK; b MRC Integrative Epidemiology Unit, University of Bristol, Bristol BS8 2BN, UK

**Keywords:** Asthma, Atopy, Suicide

## Abstract

There is emerging evidence that asthma and atopy may be associated with a higher risk of suicide. We investigated the association of asthma and atopy with mortality from suicide (*n*=32) in the Glasgow Alumni cohort, adjusting for the key confounders of socioeconomic position and smoking. We found no evidence of an association in our a priori atopy phenotypes with suicide, and there were insufficient suicides in the asthma phenotypes to draw any conclusions. In additional analyses, individuals reporting both eczema–urticaria and hay fever and those with family history of atopy were at higher risk of suicide. As these were secondary analyses and based on small numbers of events we cannot rule out chance findings. The lack of evidence in our main hypothesis may be due to the small number of suicides or reported associations between asthma and atopy may be confounded.

## Highlights

• Reported associations between asthma/atopy and suicide may be confounded.
• In our analyses we were able to adjust for socioeconomic position and smoking.
• We found no evidence of an association in our a priori atopy phenotypes with suicide.
• ‘Eczema–urticaria and hay fever’ combined and family history of atopy may be associated with higher risk of suicide.
• As these were secondary analyses and based on small numbers of events we cannot rule out chance findings.

## 1 Introduction

In 1966 Paffenbarger reported a higher percentage of allergic reaction to irritants and a consistent, “although not statistically significant”, higher percentage of asthma, among students who had died by suicide compared to controls (Paffenbarger and Asnes, 1966). Since then, a systematic review (Iessa et al., 2011) and additional papers (Timonen et al., 2001; Kuo et al., 2010; Qin et al., 2011; Loerbroks et al., 2012) have documented a higher risk of suicide ideation, attempt or death as well as depression among asthmatics and/or people with reported or measured atopy. Despite this increasing evidence, important confounders such as socioeconomic position (SEP), smoking and comorbidities were not available in all studies and so may have confounded these reported associations (Woo et al., 2012). Few studies include actual deaths by suicide (Paffenbarger and Asnes, 1966; Kuo et al., 2010; Qin et al., 2011), some with a very small number of deaths (Goodwin and Eaton, 2005). Other studies have amalgamated different asthma/atopy phenotypes making it difficult to investigate whether there is a common aetiological pathway between asthma/atopy and suicide. The Glasgow Alumni study is a historical cohort of university students, with relatively homogenous SEP in adulthood, and information available on smoking in early adulthood (McCarron et al., 1999). In addition, a subsample of the cohort provided data on depression and mental health. The aim of this study was to investigate the association of pre-defined asthma and atopy phenotypes reported in young adulthood with later suicide.

## 2 Methods

Students who attended the University of Glasgow between 1948 and 1968 were invited to an annual medical examination at the Student Health Service with approximately 50% of the students attending (*n*=15,322, 77% male, mean age 20.9 years (S.D. 4.0)) (McCarron et al., 1999). Sociodemographic and medical data were collected through a physician administered questionnaire and physical examination. Students reported “Past Medical History” of, among other conditions, having ever had i) asthma, ii) eczema–urticaria, and iii) hay fever. Eczema and urticaria were asked jointly in the questionnaire and could not be separated in the analysis. The following asthma and atopy phenotypes were initially investigated: asthma (regardless of atopic status), atopy (eczema–urticaria and/or hay fever, regardless of asthma status), and phenotypes based on the combination of these two conditions: asthma with atopy (asthma and eczema–urticaria or hay fever), asthma without atopy (asthma without eczema–urticaria or hay fever) and atopy alone (eczema–urticaria or hay fever without asthma). Secondary analyses investigated all combinations of the two atopic conditions, eczema–urticaria and hay fever, as well as family history of these conditions.

Between 2001 and 2002, members of the cohort who were still alive (*n*=8410) were contacted through a postal questionnaire and 5569 (66%) responded to this follow-up. The General Health Questionnaire (GHQ-12) (Goldberg, 1978) and history of depression in adulthood were collected in this subsample; a score of four or more on the GHQ-12 was used to indicate poor mental health. We investigated the association of asthma and atopy reported at baseline with the GHQ-12 and history of depression in adulthood in the sub-sample of questionnaire respondents. The National Health Service Central Register (NHSCR) carried out tracing of the students and provided notification of the date and cause of death, area of current residence, and emigration details. Deaths up until the end of 2012 were included. ICD-9 and ICD-10 codes were used to classify suicides into intentional self harm (ICD9: E950–E959; ICD10: X60–X84) and deaths of undetermined intent (ICD9: E980–E989, excluding E988.8; ICD10: Y10–Y34). Deaths of undetermined cause were also included in our analyses as most such deaths were suicides (Linsley et al., 2001).

Cox proportional hazards regression models were used to investigate the association of asthma and atopy with suicide using age as the time axis. The assumption of proportional hazards across exposure groups was formally tested with the Schoenfeld test. All models were adjusted for year of student examination to control for potential confounding from the change over time of disease prevalence or definitions. The final model contained terms for year of student examination, sex, height, number of siblings, birth order, father׳s social class measured with occupational class using the Registrar׳s Social class classification, body mass index (BMI) and smoking. In addition we investigated the association between poor mental health (GHQ-12 score of 4 or more) and suicide in the subsample.

## 3 Results

Students who could not be traced or who had missing exposure data were excluded from this analysis, yielding a total sample of 11,463 (8938 male, 78%) individuals among whom there were 32 suicides (29 male) up to the end of 2012. The incidence of suicide in this study was 5.52 per 100,000/year, which was substantially lower than the UK average (11.8 per 100,000/year) (ONS, 2013). The prevalence of asthma was higher in males than females (3.7% vs. 2.3%, *p*=0.001), while the prevalence of atopy was higher in females than males (12.3% vs. 9.2%, *p*<0.001). The median age of study members at the time of university medical examinations was 19.4 years (range 16–53). We found no evidence of an association of asthma and/or atopy phenotypes in young adulthood with later suicide in our a priori groups (Table 1). However, in additional analysis of secondary outcomes, individuals with a combination of eczema–urticaria and hay fever, and a family history of atopy were associated with higher risk of suicide (Table 2).

Students with missing data in the postal questionnaire were excluded, yielding a total subsample of 4746 (3568 male, 75%; 85% of the 5569 students who responded) individuals among whom there were five suicides (5 male) up to the end of 2012. A greater proportion of females reported a diagnosis of depression (19.5% vs. 14.9%, *p*<0.001), or reported poor mental health (GHQ≥4) (14.3% vs. 10.0%, *p*<0.001) compared to males. There was no evidence of an association between asthma (OR 1.10, 95%CI 0.66, 1.85, *p*=0.71), atopy (OR 0.89, 95%CI 0.66, 1.22, *p*=0.47), eczema–urticaria and hay fever (OR 0.31, 95%CI 0.04, 2.34, *p*=0.26), family history of atopy (OR 1.12, 95%CI 0.79, 1.59. *p*=0.52), or any other phenotype with a GHQ-12 score of 4 or more, or with history of depression measured in adulthood (*p*>0.05). There was weak evidence that poor mental health (GHQ-12 score of 4 or more) was associated with higher risk of suicide (model 2 adjusted HR 6.14, 95% confidence interval (CI) 0.95, 39.56, *p*=0.056).

## 4 Discussion

Contrary to previous studies (Kuo et al., 2010; Iessa et al., 2011; Qin et al., 2011) we found no evidence for our a priori hypothesis of an association of asthma and/or atopy phenotypes in young adulthood with later suicide. However, individuals with a family history of atopy or both eczema–urticaria and hay fever were associated with a higher risk of suicide. As these were secondary analyses and based on small numbers of events we cannot rule out chance findings.

The selective, relatively well-off population in our study (former university students) meant that residual confounding by SEP in adulthood is unlikely. However, their affluence may have contributed to the low incidence of suicide in this sample and so limited the power to detect clinically important associations with our a priori phenotypes. On the other hand, this is one of the only a few studies to investigate the association between asthma or atopy with death from suicide, as opposed to suicidal ideation or attempted suicide (Paffenbarger and Asnes, 1966; Goodwin and Eaton, 2005; Kuo et al., 2010; Qin et al., 2011, 2013), an approach recommended in a recent review on this topic (Goodwin, 2012).

A small number of suicides in this study limited the power to detect associations increasing our chance of reporting type II errors. In one of our a priori hypotheses there were 5 suicides from 1132 individuals reporting atopy compared with 27 suicides from 10331 individuals not reporting atopy. A retrospective power calculation performed using stata (StataCorp, 2013) calculated we had 25% power at 5% significance level to detect an HR of 1.69 or more. To achieve 80% power at a 5% significance level it would require a total sample size of approximately 34,000 individuals, with equal numbers in the atopy and no atopy groups. Alternatively, given our sample size and the number of suicides in individuals with no atopy, at 80% power and a 5% significance level we were only able to detect an effect size approximately twice the size of that observed (an HR3.23 compared with an HR1.69 observed for atopy analysis). Previous studies have reported that asthma or atopy is associated between a 1.2 and 3.5 times higher odds of suicidal ideation, suicide attempts or completed suicide (Goodwin and Eaton, 2005; Messias et al., 2010; Qin et al., 2011).

A potential source of bias could be due to only half of the students who were invited to the medical examination actually attending. If those who did not attend the medical examination had a higher prevalence of asthma/atopy, and there were more suicides in this group of individuals, then the results of this study would be biased towards the null. This limitation is also relevant to the postal questionnaire where individuals with more severe asthma, atopy or depression may be less likely to respond. This may explain why we failed to find evidence of an association as strong as has been previously reported and thus a lack of evidence supporting our a priori hypotheses.

We report an association between family history of atopy with a higher risk of suicide which supports previous work reporting an association between family history of atopy and depression (Timonen et al., 2003). Twin studies have suggested a shared genetic risk for atopic and depressive symptoms (Wamboldt et al., 2000). However, a shared genetic risk would not fully explain our results as it appears a family history of atopy is a stronger risk factor than having atopy yourself. It may be that atopy conveys risk, but its treatment, in particular with intranasal or inhaled corticosteroids reduces the risk (Woo et al., 2011). In addition, exposure to molecular mediators of atopy, or atopy medication during pregnancy may influence brain development and increase the risk of suicide in the offspring. A limitation of this study is that medication data or data on other physical diseases were not included in the analyses but may be confounding the results. There was only 1 suicide in the asthma group which limited the conclusions that could be drawn.

Our finding that individuals with both eczema–urticaria and hay fever are at a 9 fold higher risk of suicide is consistent with other reports of only severe atopy being associated with suicide in a Danish record-linkage study (Qin et al., 2011) and a dose-response association between air pollen counts and suicide (Qin et al., 2013). This type of dose-response association has also been reported between the severity of asthmatic symptoms and subsequent suicide risk (Kuo et al., 2010). However, in our analysis, the finding was based on only two suicides and there was no association with other measures of mental health, so must be interpreted with extreme caution.

Several mechanisms could explain an association between asthma and/or atopy with suicide: i) confounding due to cigarette smoking, child and/or adulthood deprivation and other demographic characteristics. We could account for some of these in our analysis and through the study design. In addition, depression or other mental health comorbidities may be confounders if they are not on the casual pathway; ii) hopelessness experienced due to suffering a lifelong chronic condition (Goodwin and Buka, 2008); and, iii) a common risk factor, related to inflammatory processes, to which both conditions are related (Miller et al., 2009). Our results point to processes related specifically to atopic conditions. As noted above an association with family history of atopy could suggest shared genetic or biological traits.

This study adds to the growing body of research investigating the association among asthma, atopy and suicide. Our study included deaths from suicide rather than ideation and could account for some relevant confounders. Replication of these findings in other populations with comprehensive information on confounders, particularly life course mental history, and biological markers of potential aetiological mechanisms is necessary.

## Figures and Tables

**Table 1:** Hazard Ratio (HR) and 95% Confidence Interval (CI) of a priori exposures with death from suicide.

Exposure	Individuals (%)	Suicides (*n*)	Model 1 HR (95% CI)	Model 2 HR (95% CI)
Asthma (with or without atopy)^a^	387 (3.4)	1	0.92 (0.13–6.76)	0.92 (0.13–6.73)
Atopy (with or without asthma)^a^	1132 (9.9)	5	1.68 (0.65 –4.36)	1.69 (0.65–4.40)
Asthma and atopy phenotypes				

No asthma, no atopy (reference group)	10089 (88.0)	27	1.00	1.00
Asthma with atopy	145 (1.3)	1	2.50 (0.34–18.40)	2.55 (0.35–18.89)
Asthma without atopy	242 (2.1)	0	–	–
Atopy without asthma	987 (8.6)	4	1.51 (0.53–4.32)	1.51 (0.53–4.34)

Model 1 adjusted for year of examination. Model 2 also adjusted for sex, height, number of siblings, birth order, fathers social class, body mass index and current smoking.

a There were a total of 32 suicides in the cohort of 11,463.

**Table 2:** Hazard Ratio (HR) and 95% Confidence Interval (CI) of secondary exposures with death from suicide.

Exposure	Individuals (%)	Suicides (*n*)	Model 1 HR (95% CI)	Model 2 HR (95% CI)
Hay fever only	584 (5.1)	2	1.26 (0.30–5.29)	1.22 (0.29–5.15)
Eczema–urticaria only	352 (3.1)	0	–	–
Eczema–urticaria and hay fever	75 (0.7)	2	10.02 (2.39–42.07)	9.51 (2.24– 40.43)
Family history of atopy (with or without atopy themselves)	693 (6.1)	6	3.56 (1.46–8.65)	4.25 (1.73–10.44)
Family history of asthma (with or without asthma themselves)	551 (4.8)	3	2.08 (0.63–6.84)	2.44 (0.74–8.06)

Model 1 adjusted for year of examination. Model 2 also adjusted for sex, height, number of siblings, birth order, fathers social class, body mass index and current smoking. There were a total of 32 suicides in the cohort of 11,463. All analyses included the 11,463 individuals.

## References

[bib1] Goldberg D. (1978). Manual of the General Health Questionnaire.

[bib2] Goodwin R.D. (2012). Asthma and suicide: current knowledge and future directions. Current Psychiatry Reports.

[bib3] Goodwin R.D., Buka S.L. (2008). Childhood respiratory disease and the risk of anxiety disorder and major depression in adulthood. Archives of Pediatrics and Adolescent Medicine.

[bib4] Goodwin R.D., Eaton W.W. (2005). Asthma, suicidal ideation, and suicide attempts: findings from the Baltimore epidemiologic catchment area follow-up. American Journal of Public Health.

[bib5] Iessa N., Murray M., Curran S., Wong I. (2011). Asthma and suicide-related adverse events: a review of observational studies. European Respiratory Review.

[bib6] Kuo C.J., Chen V.C.H., Lee W.C., Chen W.J., Ferri C.P., Stewart R., Lai T.J., Chen C.C., Wang T.N., Ko Y.C. (2010). Asthma and suicide mortality in young people: a 12-year follow-up study. American Journal of Psychiatry.

[bib7] Linsley K., Schapira K., Kelly T. (2001). Open verdict v. suicide-importance to research. The British Journal of Psychiatry.

[bib8] Loerbroks A., Herr R.M., Subramanian S., Bosch J.A. (2012). The association of asthma and wheezing with major depressive episodes: an analysis of 245 727 women and men from 57 countries. International Journal of Epidemiology.

[bib9] McCarron P., Davey Smith G., Okasha M., McEwen J. (1999). Life course exposure and later disease: a follow-up study based on medical examinations carried out in Glasgow University (1948–68). Public Health.

[bib10] Messias E., Clarke D.E., Goodwin R.D. (2010). Seasonal allergies and suicidality: results from the National Comorbidity Survey Replication. Acta Psychiatrica Scandinavica.

[bib11] Miller A.H., Maletic V., Raison C.L. (2009). Inflammation and its discontents: the role of cytokines in the pathophysiology of major depression. Biological Psychiatry.

[bib12] ONS (2013). Suicides in the United Kingdom, 2011.

[bib13] Paffenbarger R.S., Asnes D.P. (1966). Chronic disease in former college students 3. precursors of suicide in early and middle life. American Journal of Public Health and the Nations Health.

[bib14] Qin P., Mortensen P.B., Waltoft B.L., Postolache T.T. (2011). Allergy is associated with suicide completion with a possible mediating role of mood disorder – a population-based study. Allergy.

[bib15] Qin P., Waltoft B.L., Mortensen P.B., Postolache T.T. (2013). Suicide risk in relation to air pollen counts: a study based on data from Danish registers. BMJ Open.

[bib16] StataCorp (2013). Stata Statistical Software: Release 13.

[bib17] Timonen M., Hakko H., Miettunen J., Karvonen J.T., Herva A., Rasanen P., Koskinen O., Zitting P. (2001). Association between atopic disorders and depression: findings from the northern Finland 1966 birth cohort study. American Journal of Medical Genetics.

[bib18] Timonen M., Jokelainen J., Herva A., Zitting P., Meyer-Rochow V.B., Rasanen P. (2003). Presence of atopy in first-degree relatives as a predictor of a female proband׳s depression: results from the Northern Finland 1966 Birth Cohort. Journal of Allergy and Clinical Immunology.

[bib19] Wamboldt M.Z., Hewitt J.K., Schmitz S., Wamboldt F.S., Rasanen M., Koskenvuo M., Romanov K., Varjonen J., Kaprio J. (2000). Familial association between allergic disorders and depression in adult Finnish twins. American Journal of Medical Genetics.

[bib20] Woo J.M., Gibbons R.D., Qin P., Komarow H., Kim J.B., Rogers C.A., Mann J., Postolache T.T. (2011). Suicide and prescription rates of intranasal corticosteroids and nonsedating antihistamines for allergic rhinitis: an ecological study. The Journal of Clinical Psychiatry.

[bib21] Woo J.M., Gibbons R.D., Rogers C.A., Qin P., Kim J.B., Roberts D.W., Noh E.S., Mann J.J., Postolache T.T. (2012). Pollen counts and suicide rates. Association not replicated. Acta Psychiatrica Scandinavica.

